# Preoperative Robotics Planning Facilitates Complex Construct Design in Robot-Assisted Minimally Invasive Adult Spinal Deformity Surgery—A Preliminary Experience

**DOI:** 10.3390/jcm13071829

**Published:** 2024-03-22

**Authors:** Martin H. Pham, Nicholas S. Hernandez, Lauren E. Stone

**Affiliations:** Department of Neurosurgery, UC San Diego School of Medicine, University of California, San Diego, CA 92037, USAlestone@health.ucsd.edu (L.E.S.)

**Keywords:** adult spinal deformity (ASD), minimally invasive surgery (MIS), spinal robotics, complex construct design, preoperative robotics planning, Mazor X Stealth Edition

## Abstract

(1) **Background:** The correction of adult spinal deformity (ASD) can require long, complex constructs with multiple rods which traverse important biomechanical levels to achieve multi-pelvic fixation. Minimally invasive (MIS) placement of these constructs has historically been difficult. Advanced technologies such as spinal robotics platforms can facilitate the design and placement of these constructs and further enable these surgical approaches in MIS deformity surgery. (2) **Methods:** A retrospective study was performed on a series of ASD patients undergoing MIS deformity correction with ≥eight fusion levels to the lower thoracic spine with preoperative robotic construct planning and robot-assisted pedicle screw placement. (3) **Results:** There were 12 patients (10 female, mean age 68.6 years) with a diagnosis of either degenerative scoliosis (8 patients) or sagittal imbalance (4 patients). All underwent preoperative robotic planning to assist in MIS robot-assisted percutaneous or transfascial placement of pedicle and iliac screws with multiple-rod constructs. Mean operative values per patient were 9.9 levels instrumented (range 8–11), 3.9 interbody cages (range 2–6), 3.3 iliac fixation points (range 2–4), 3.3 rods (range 2–4), 18.7 screws (range 13–24), estimated blood loss 254 cc (range 150–350 cc), and operative time 347 min (range 242–442 min). All patients showed improvement in radiographic sagittal, and, if applicable, coronal parameters. Mean length of stay was 5.8 days with no ICU admissions. Ten patients ambulated on POD 1 or 2. Of 224 screws placed minimally invasively, four breaches were identified on intraoperative CT and repositioned (three lateral, one medial) for a robot-assisted screw accuracy of 98.2%. (4) **Conclusions:** Minimally invasive long-segment fixation for adult spinal deformity surgery has historically been considered laborious and technically intensive. Preoperative robotics planning facilitates the design and placement of even complex multi-rod multi-pelvic fixation for MIS deformity surgery.

## 1. Introduction

In adults aged 65 years and older, adult spinal deformity (ASD) may have a prevalence of up to 68%, and may lead to chronic back pain and neurologic deficits, in turn leading to disability [1]. Self-image, pain, and disability are found to be the most common drivers for patients to pursue surgical correction when conservative treatment measures have failed [2]. In a preoperative survey of adults planning to undergo ASD correction, 66% of patients anticipated a highly successful operation, with an average expected reduction in pain of 71% [3]. Surgeon expectations for deformity correction largely focus on functional improvement and radiographic correction to prevent future disability [4]. While there may be a mismatch in the primary expectations for surgical correction, there is a shared goal to achieve a robust functional outcome, reduce pain, and prevent future complications. As such, ASD correction strategies continue to evolve to maximize these benefits. More surgical procedures are carried out in a minimally invasive fashion when able, and there is now Level 3 evidence which shows that patients have a positive perspective of minimally invasive surgery (MIS) in the spine, and prefer to have MIS spine surgery when able [5].

MIS techniques are well described in the treatment of degenerative disease, trauma, and ASD [6]. Compared to open procedures, MIS approaches reduce estimated blood loss (EBL), minimize tissue trauma, and shorten length of stay (LOS), while also restoring sagittal and coronal balance, promoting fusion, and decompressing the neural elements [7]. Patient-reported outcomes such as self-image, mental health, and satisfaction may also improve earlier in patients undergoing MIS compared to open ASD surgery [8]. These benefits have driven the creation of novel techniques for several contemporary open approaches, as surgical centers continue to bring more optimized outcomes to their patients.

As MIS approaches become further optimized and more familiar, these techniques may be applied to pathology not previously amenable to MIS, such as complex ASD. In these cases, effective and sustainable correction may require complex constructs. These advanced construct designs employ multiple rods and various loading distribution techniques to increase pillar support, bolster vulnerable osteotomy sites, enhance rigidity, and promote fusion in order to restore sagittal balance, improve load absorption, increase posterior column mobility, and restore lumbar lordosis (LL) [9,10,11,12,13]. Traditional open surgical techniques have previously required large incisions through the paraspinal fascia, with near total dissection of the paraspinal musculature to expose the posterior bony elements of the spine to implement these constructs. In MIS techniques, preservation of the posterior fascia and paraspinal muscles aims to reduce surgical trauma, but comes at the expense of the visualization of several landmarks when planning bony fixation, which in turn limits its applicability to larger and complex construct designs in ASD. A carefully planned preoperative design is therefore critical for desired postoperative correction [14]. This is especially important in MIS constructs where the spine is less exposed and the ability to see or modify these plans extemporaneously is reduced. The placement of long-segment MIS constructs can also be technically demanding, especially for surgeons less familiar with these techniques. In order to increase intraoperative confidence and reduce rates of “conversion to open” in MIS cases, a thorough and accurate preoperative plan for MIS correction in ASD may assist surgeons when offering MIS to patients with ASD. Barriers to adoption include concerns over appropriate tulip head alignment, minimally invasive tower management with collisions at the lumbosacral junction, and the minimally invasive passage of a long rod across multiple fixation levels. This report aims to describe a series of cases in which preoperative robotics planning software was used to address these points while designing complex constructs to correct ASD to be performed through an MIS approach.

A more recent advancement in the armamentarium of technology in ASD surgery is the inclusion of spinal robotic platforms. Robotic systems are now used in several spinal procedures, and were recently shown to provide a very high degree of screw accuracy and safety when compared to previous conventional techniques [15]. These systems not only include robotic arm and intraoperative navigation technology, but now also provide for simulation and planning software. The development of robotics systems with their requisite preoperative software planning enables comprehensive preoperative assessments and allows for not only straightforward minimally invasive placement of long-segment instrumentation but the design of complex construct designs that involve multi-rod and multi-pelvic fixation. We report here a case series of adult spinal deformity patients treated with robot-assisted minimally invasive surgical techniques and describe the feasibility of these techniques for wider adoption.

## 2. Methods

This study describes a retrospective series of patients at a single academic center who underwent minimally invasive correction of ASD with robotic assistance, with preoperative planning of complex constructs performed by a single attending neurosurgeon (M.H.P). In our series, ASD was defined as pelvic tilt (PT) > 25°, pelvic incidence minus lumbar lordosis (PI-LL) > 10°, sagittal vertical axis (SVA) > 5 cm, or coronal Cobb angle > 20°. A complex construct was defined as ≥8 fusion levels with an upper instrumented level (UIV) into the lower thoracic spine. Data points collected included demographic characteristics, operative time, estimated blood loss (EBL), pedicle screw accuracy, radiographic alignment, inpatient stay metrics, and complications.

This study was reviewed and approved by the Institutional Review Board of the University of California San Diego (# 210617, approved on 23 January 2023), and all patients consented to participation in research prior to enrollment.

### 2.1. Preoperative Robotic Construct Design

**Figure 1:**
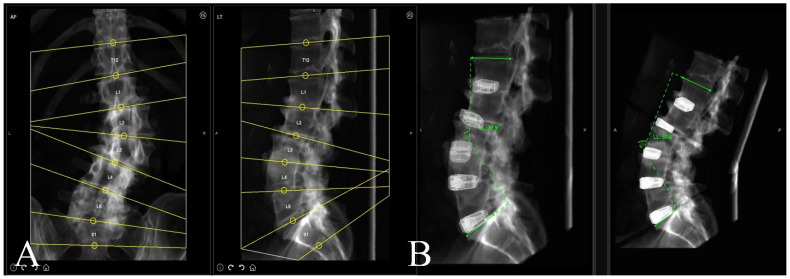
(**A**) Robotic software automatic segmentation of each vertebral level as a separate independent volume to allow for both screw fixation and interbody implant planning. (**B**) Sagittal simulation of interbody cage placement with movement of each individual vertebral segment to assess if appropriate sagittal correction can be achieved with minimally invasive placement of interbodies.

**Figure 2:**
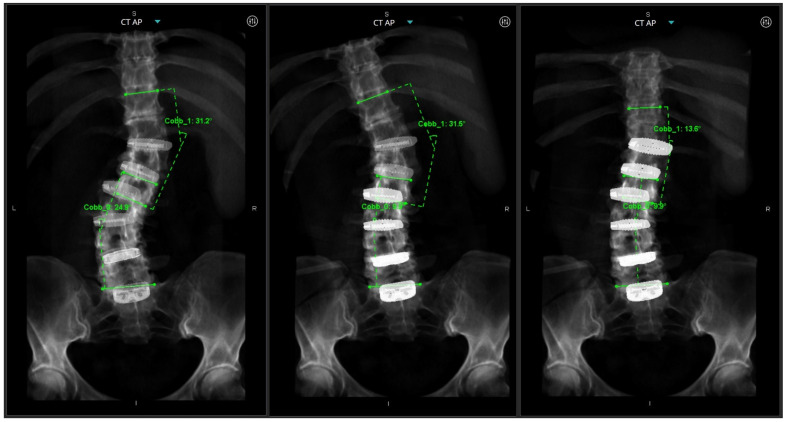
Coronal simulation of interbody cage placement to assess if minimally invasive placement of interbodies can effectively correct fractional and main coronal curve deformities through movement at each vertebral segment.

**Figure 3:**
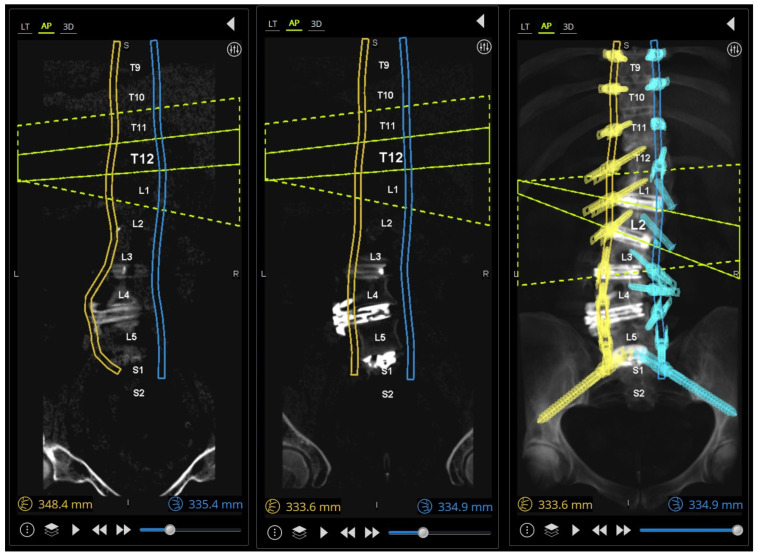
Rod simulation as each pedicle screw is placed in the software. (**left**) The yellow left-sided rod geometry is not amenable to planar cranial–caudal placement when screws are planned in “perfect ideal” lateral-to-medial trajectories; (**middle**,**right**) the yellow left-sided rod geometry is now planar after adjusting screw trajectories to be straighter, treating pedicles more as bone for fixation in context of the entire construct, rather than individual perfect bone columns to maximally fill.

**Figure 4:**
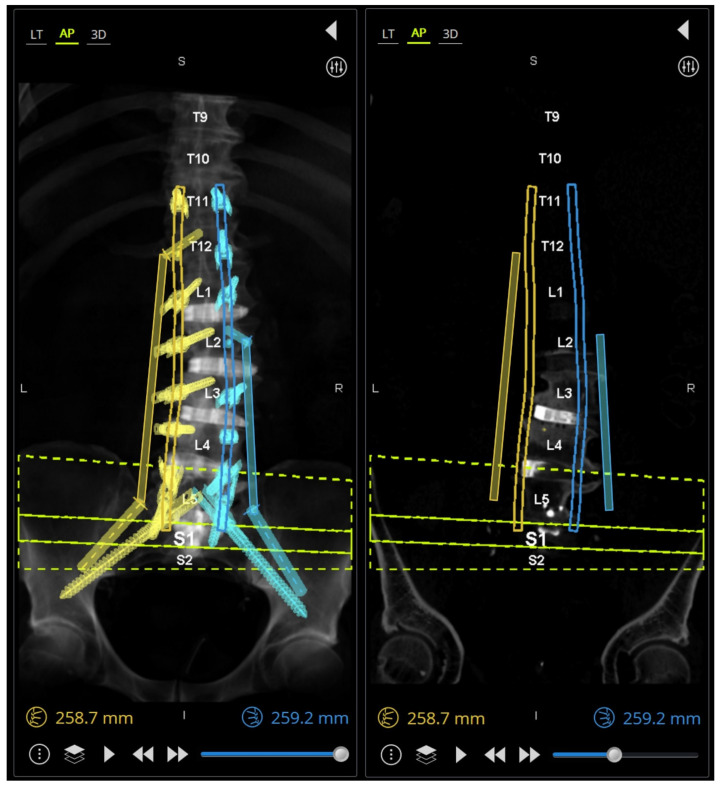
A multiple-rod construct with four iliac screws. Due to the limitations of dual-headed screws or dominos in MIS deformity techniques, satellite (accessory or kickstand) rods are currently designed to connect pedicle screws to traditional iliac screws without direct connection to the main rod.

A preoperative thin-cut CT scan is obtained and loaded into the planning software associated with the spinal robotics system (Mazor X Robotics Planning Software Version 5.0 with X-Align, Medtronic Sofamor Danek, Minneapolis, MN, USA) (Figure 1A). Interbody cages are then planned and simulated. Of note, the software assumes full movement of the chosen segment based on cage geometry and marked endplate surfaces, and under-correction of the simulation may oftentimes be needed due to rigid deformities or facet ankylosis and hypertrophy (Figure 1B and Figure 2). With this correction provided, pedicle and iliac screws are then planned from the UIV to S2 and bilateral rods are simulated after each screw to confirm appropriate planar alignment (Figure 3). Adjustments to the trajectories of the pedicle screws can be made to ensure easy subfascial passage. Satellite accessory rods are planned using lateral-to-medial screw trajectories with positions outside of the main rod. While open surgical techniques allow for satellite rod placement with dual-headed screws or rod–rod domino connectors, the lack of direct visualization in MIS makes these strategies prohibitive and therefore satellite rods are usually not able to be directly connected to the main rod (Figure 4).

**Figure 5:**
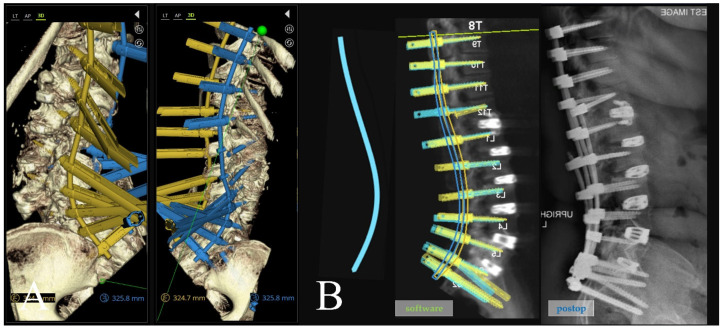
(**A**) Three-dimensional reconstruction of the simulated rod, pedicle screws, and minimally invasive extension towers. Note the focus at the L4–S2 levels where improper planning may lead to these respective screw towers colliding and potentially blocking subsequent screw placement. Small adjustments can be made in real time in this view to resolve potential collisions. (**B**) (**left**) A patient-specific rod geometry designed with predictive parameters; (**middle**) the robotic plan with screws in place aligned to the simulated correction; (**right**) postoperative standing lateral X-ray showing good apposition to rod geometry and plan.

The extension towers from L4 to S2 need to be reviewed in detail because of their converging trajectories (Figure 5A), and minor adjustments can be made to the pedicle screw targeted positioning in the sagittal plane to avoid tower collisions at the skin level. If a patient-specific rod (PSR) is being used, screw planning can be performed to ensure rod geometries are appropriately similar and aligned after the simulated correction (Figure 5B).

### 2.2. Operative Technique

For Stage 1, placement of all interbody cages in this series was carried out using an anterior-to-psoas (ATP) technique also known as an oblique lumbar interbody fusion (OLIF) for levels above L5-S1, as well as a lateral anterior lumbar interbody fusion (ALIF) at L5-S1 as a first stage of surgery. The patient is first positioned in the right lateral decubitus position with the left side up. Because there may be a certain degree or rotational deformity that requires bed rotation, the patient is generously taped and secured to the bed. This also allows for the use of intraoperative navigation while minimizing inaccuracies of the navigation system. Incisions are then marked depending on the incisional access to the respective disc spaces; for multiple interbody placement, this may usually require 2–3 separate incisions, with each incorporating at least 2 interbody levels. For interbody levels above L5-S1, the retroperitoneal space is accessed after blunt dissection through the abdominal wall in line with the muscular fibers, and the disc space is palpated at the anterior border of the psoas. The peritoneal contents are carefully maintained in a forward and anterior position to avoid the great vessels based on knowledge of their position through evaluation on preoperative imaging. Minimally invasive retractor systems are then placed with discectomy, disc prep, and trialing to follow. If the anterior longitudinal ligament is released, then interbody fixation screws are placed. For the L5-S1 level, a retroperitoneal approach with access of the disc space between the great vessels is performed similar to the surgical corridor in a supine ALIF, but with the patient in lateral position. Once retractor blades are set with careful protection of the left common iliac vein (LCIV), discectomy and trialing proceed in a similar fashion with subsequent placement of an ALIF footprint cage. Usually, only one interfixated screw is placed to allow for further lordosis, realignment, or correction of the fractional curve from the posterior stage if needed.

Closure of the anterior stage of the procedure proceeds in the usual fashion. Following Stage 1, standing radiographs are taken to determine the degree of achieved correction, residual or new radiculopathy, and if additional coronal or sagittal balance is necessary in the second stage of surgery. As such, this interval provides a secondary opportunity to adjust the preoperative planning design for the final construct.

**Figure 6:**
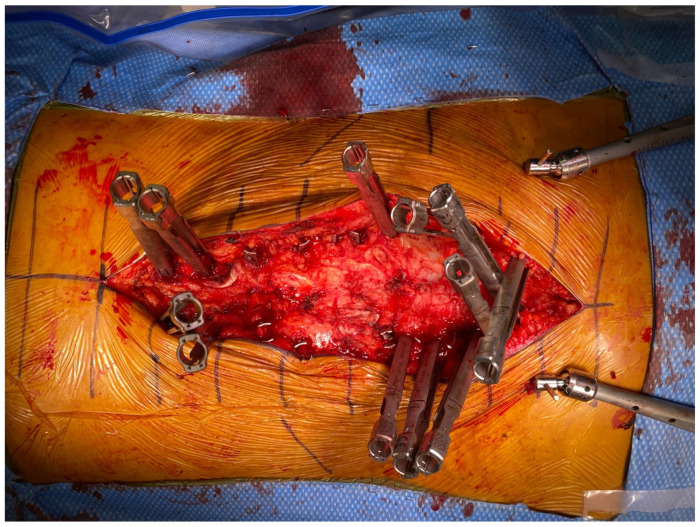
Intraoperative view showing a single skin incision with transfascial placement of all screws. Note extension towers were left off the L1-4 pedicle screws to facilitate visualization for subsequent mini-open posterior column osteotomies.

Stage 2 of the procedure is next carried out on the second operative day with the patient in the prone position. To minimize the introduction of movement error that could affect robotic accuracy, we add circumferential tape during positioning at the best pad below the axilla and at the distal buttocks. Anesthesia also administers muscle relaxant after monitoring baselines to limit delayed sag or patient movement during instrumentation placement. The robotics system is registered to the patient and screws are placed transfascially through a single midline skin incision or percutaneously through multiple incisions if the patient has a high BMI (Figure 6). All techniques are performed using what we term “light-touch surgery”, whereby all instruments pass down perfectly and smoothly coaxial to the robotic arm’s end effector to minimize its deflection; any sticking is treated with irrigation and xeroform. Screws are placed in a sequence that is proximal (UIV) to distal (S1), and then all S2-alar-iliac (S2AI) or traditional iliac screws are placed last. This is to ensure maximum accuracy with the screws furthest away from the system most vulnerable to movement error, and because the placement of iliac screws generates an incredible amount of torque that can introduce error into the system. If there is any concern of error, robot and navigation checks are performed or the patient is re-registered with updated C-arm X-rays out of an abundance of caution.

**Figure 7:**
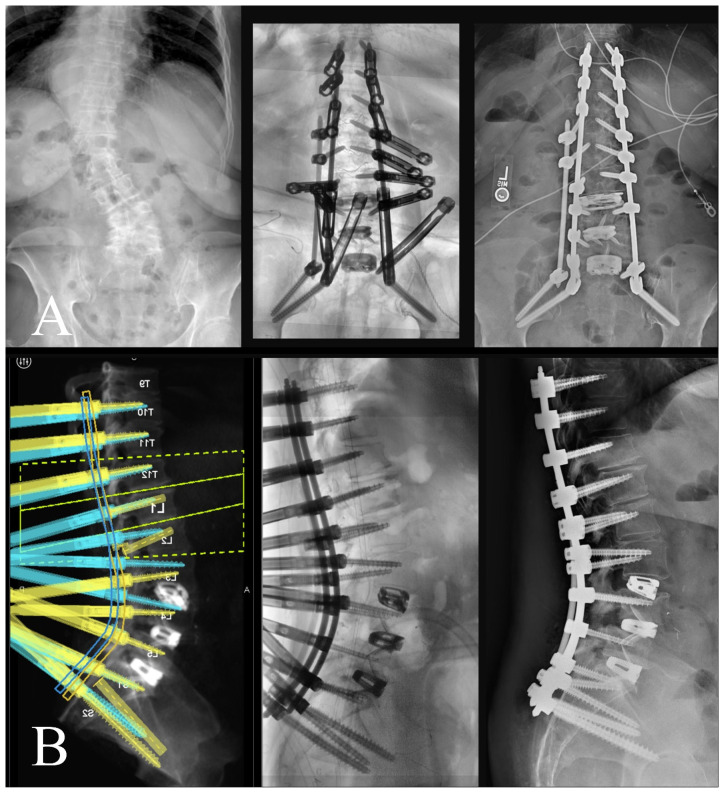
(**A**) (**left**) Preoperative AP X-ray; (**middle**) intraoperative AP long film showing appropriate coronal correction; (**right**) postoperative standing AP X-ray. (**B**) (**left**) Preoperative robotic plan with patient-specific rod geometry; (**middle**) intraoperative sagittal long film with appropriate sagittal correction; (**right**) postoperative standing lateral X-ray.

An intraoperative CT scan with a navigation frame attached to the patient is then obtained as a confirmation scan and to allow for navigated repositioning of any screws that are needed. A navigated burr is then used through the existing transfascial or percutaneous incisions to decorticate and drill out all facet joint levels that do not have anterior interbody fusions. These decorticated pockets are then packed with the bone graft of choice for the surgery. If needed, a mini-open exposure is performed for posterior column osteotomies (PCOs) to allow for further lordosis or scoliosis curve correction. Rods are then passed using a minimally invasive technique with rod passage inserters. While this historically has carried the possibility of great difficulty, the enabling technologies of planar screw planning has allowed this to proceed in very routine fashion. Satellite rods are first secured and locked into position so that their minimally invasive towers can be removed from the working airspace over the wound and any distractive techniques are completed if they are functional kickstand rods [16]. Placement of both main rods then follows. Acceptable alignment is then confirmed using a long film or a series of stitched X-rays (Figure 7). Closure proceeds in the usual fashion after all set screws are secured and towers removed.

## 3. Results

There were 12 patients included in the study (10 female), with a mean age of 68.6 years (range 60–77) and either a diagnosis of degenerative scoliosis (8 patients) or sagittal imbalance (4 patients). All patients underwent minimally invasive robot-assisted percutaneous or transfascial placement of pedicle and iliac screws. Baseline demographic characteristics of the cohort are shown in Table 1. The operative parameters of the cohort are shown in Table 2 with radiographic correction shown in Table 3.

Mean operative values per patient were 9.9 levels instrumented (range 8–11), 3.9 interbody cages (range 2–6), 3.3 iliac fixation points (range 2–4), 3.3 rods (range 2–4), and 18.7 screws (range 13–24). Estimated blood loss was 254 cc (range 150–350 cc) with no patients requiring intraoperative blood transfusions. A total of 224 screws were placed minimally invasively with robotic assistance, with four breaches identified on intraoperative CT and repositioned (three lateral, one medial) for a screw accuracy of 98.2%.

Mean operative skin time for the Stage 2 robot-assisted posterior instrumentation was 347 min (range 242–442 min). Sub-analysis showed six patients who underwent minimally invasive placement of screw fixation only had mean operative times of 305 min, whereas the other six patients who also underwent mini-open laminectomies or posterior column osteotomies (mean 4 levels, range 3–6) had mean operative times of 374 min.

Mean improvement in spinopelvic alignment were sagittal vertical axis (SVA) −5.2 cm (range −20.3 to 4.5 cm), pelvic tilt (PT) 10.8° (range 1–23°), and pelvic incidence–lumbar lordosis (PI-LL) mismatch 21.9° (range −5° to 47°). Eight patients with scoliosis showed improvements in their coronal Cobb angles of 27.3° (range 19–43°). Ten patients ambulated within the first 2 postoperative days. The mean LOS was 5.8 days (range 4–10) and there were no ICU admissions.

Mean follow-up was 21.7 months (range 6–42). There were two reoperations for proximal junctional failure (PJF) (patients 2 and 3). One patient presented with early radiographic evidence of proximal junctional kyphosis (PJK) after a mechanical fall but is currently asymptomatic (patient 9). One patient suffered aspiration pneumonia between her first- and second-stage surgeries (patient 5), resulting in a prolonged hospital stay for respiratory recovery and persistent drug fevers. There were no instances in follow-up of surgical site infections, new neurologic deficits, pseudarthrosis, or implant failure.

## 4. Discussion

While the benefits of MIS for degenerative spine surgery have been well studied, descriptions of its application in deformity correction have required a closer assessment of these techniques’ effectiveness for instrumentation accuracy, achieved fusion, and improvement in coronal and sagittal balance when applied to ASD correction. Several retrospective reviews have suggested that MIS approaches for ASD provide comparable outcomes of these parameters when compared to open surgery, with numerous other reported benefits consistent with an MIS profile [17,18,19,20,21]. Recognizing certain limitations of MIS approaches in severe or stiff deformities, treatment algorithms were also developed to guide decision making for patients who could benefit from minimally invasive techniques [22]. While this tool has been shown to be useful and reliable, these decision algorithms have not yet included the incorporation of preoperative robotics software for construct design and planning.

This is an important adjunct to patient selection and operative planning, as the inclusion of these tools may increase the pool of patients in which MIS deformity correction may be considered. More recent reports have shown that in cases of even marked deformity, MIS techniques have shown to be quite effective while still benefiting from reduced complication profiles [23]. While these data may indicate that MIS approaches are feasible to correct ASD, they may not underscore the intraoperative limitations and challenges of applying such an approach. Anticipation of the challenges to applying MIS in ASD correction, such as tower collision, fixation of satellite rods, subfascial passage of rods, etc., is imperative for bringing these techniques into regular practice. Our report therefore advocates for preoperative planning of MIS constructs using robotic software to design these constructs in three-dimensional space, but also to modify screw and rod trajectories as intraoperative collisions and conflicts are anticipated. For example, Figure 7 illustrates a case in which two right-sided pedicle screws are preoperatively selected to affix to a satellite rod, rather than the main rod, with pre-adjusted trajectories of these screws allowing for easy intraoperative passage of right-sided rods. Loading pre-planned screw trajectories into the surgical robot ensures accurate and streamlined transfascial placement, accounting for previous components of the case during Stage 1 when interbody cages are placed. Also illustrated in Figure 7 is the omission of a of the left L5 pedicle screw, to avoid tower collision when lordosis correction is achieved. Tower collision at the lumbosacral junction is a common spatial limitation in the operative workspace and may be difficult to anticipate as lordosis correction is achieved during open surgery. This demonstrates how preoperative planning software may aid in anticipating intraoperative spatial limitations of the workflow and permits adjustments of the construct design to yield a surgical plan which achieves an optimal surgical correction but is also technically feasible through an MIS approach. Prior studies showing use of robotics in adult spinal deformity have mostly relied on accuracy of screws or placement of S2-alar-iliac pelvic fixation, which highlights the need to expand upon the benefits of robotics use specifically during this planning stage [24].

Other groups and institutions have implemented various techniques to improve the quality of the extent of preoperative planning in deformity correction to increase operative confidence, increase screw accuracy, and decrease intraoperative fluoroscopy time and surgical complications. One such adjunct is the use of 3D-printed anatomical models for preoperative planning. In a systematic review, these 3D-printed models were shown to increase screw accuracy and improve correction, though they could be associated with significant production costs and time [25]. Additionally, these models are generally used for planning in open surgical correction. Further, while these models may be quite useful in understanding preoperative deformity in order to plan instrumentation, they do not allow for a dynamic understanding of screw trajectory as the deformity parameters change intraoperatively. Again, our series here demonstrates that preoperative robotics software permits a continuous assessment of deformity correction as implants, screws, and rods are planned into the final construct.

Another method of preoperative planning used in ASD is machine learning software to generate patient-specific rods (PSR). This software analyzes the current deformity, then generates a rod with an appropriate length and contour to achieve the final desired correction. A series of 20 patients undergoing ASD correction with preoperative planning for PSRs showed that this software enabled accurate and feasible correction, though in open surgery [26]. Importantly, not all cases included two-stage correction with the use of anterior lumbar interbody fusion (ALIF) for interbody cages. Further, this series found that distal junctional failure was associated with the use of PSRs and was often related to the absence of interbody grafts at the lumbosacral junction. This may emphasize the importance of preoperative planning software which simultaneously calculates and plans for necessary interbody graft inclusion and the anticipated contour of the final fixation rods. Use of these rods also requires careful and thoughtful prone positioning and the use of Smith-Peterson osteotomies during the case to achieve lordotic correction to allow fixation of the PSR. The preoperative planning workflow presented here instead includes more complex construct designs which include multiple interbody cages, often across important junctional levels, leading to deformity correction which takes place prior to rod placement. In turn, the workflow presented here may be more amenable to an MIS approach in which deformity correction prior to rod passage requires less posterior bony manipulation in order to fix the PSR into the final construct, and includes pre-planned interbody grafts to minimize the risk of junctional failure and reoperation.

In addition to the skillset of lateral access surgery which enables the majority of spinal deformity corrections in MIS surgery, a major barrier to the adoption of MIS deformity surgery is the subsequent requisite long-segment posterior fixation requiring multiple pedicle and iliac screws to be placed minimally invasively, followed by the passage of several long-segment rods. Three-dimensional navigation technologies have reduced these difficulties by providing real-time computer-aided visualization of anatomy in the operating room for placement of these implants [15,27]. We demonstrate here that robotics platforms, with their ability to preoperatively design constructs, can further reduce this barrier to adoption by providing the ability to preoperatively place pedicle and iliac screws for subsequent execution with the robotic arm in the operating room. This was shown to be feasible even for multiple rod placements with multiple iliac fixation points, with mean operating room times of 5 h and 47 min and a screw accuracy of 98.2% across a cohort with a mean of 9.9 levels fused to the lower thoracic spine. While the use of robotics systems has been described for the treatment of adult spinal deformity, the vast majority of these have been for open surgery or descriptions of S2-alar-iliac screw placement [24]. MIS placement of screws in short-segment degenerative pathologies has also been well reported [15,27,28], but their use for long-segment complex deformity correction has been described less frequently. Our series herein thereby serves to illustrate that long-segment constructs can be designed for MIS correction of ASD. The software here aids the design of screw numbers, trajectory, anticipated interbody grafts, and fixation rods. These designs are therefore created within the constraints of an MIS approach, and can be modified preoperatively to be tailored to each patient’s anatomy and how each patient’s anatomy is projected to change following correction. Placement interbody grafts, followed by pedicle screws and posterior column osteotomies, inherently adjust the spinopelvic parameters of the patient. In turn, this may complicate rod passage, and may limit further lordosis correction by rod bending once all rods are seated. Robot-assisted calculation of these changes with adjustments in construct design helps to ensure that all steps of posterior fixation are practically feasible, but also that fine manipulations to the final construct are allowed through the MIS approach to achieve the desired final result.

Our cohort’s final radiographic parameters show the successful realignment of sagittal parameters as an endpoint even in cases of marked sagittal imbalance (patient 6, SVA 21.0 cm; patient 12, SVA 20.4 cm) and mean improvement in coronal Cobb measurements of 27.3° for patients with degenerative scoliosis, highlighting the success of MIS techniques in appropriately selected patients. While the success of open spinal deformity is well established [17,18,19], complication profiles can differ, and prior studies have demonstrated the benefits of minimally invasive approaches to reduce intraoperative and postoperative complications and hospital stay lengths [22]. Of note, there were no wound, neurologic, or implant-related complications in our series, which is consistent with these prior studies. Here, we achieved optimal radiographic correction outcomes, with similar complication profiles to that of the open literature. Again, these results emphasize that careful preoperative construct planning in select patients allows for comparable outcomes in ASD correction through an MIS approach when compared to open robotic-assisted techniques.

While the majority of MIS deformity correction in ASD relies on anterior realignment through the lateral placement of multiple interbody cages, subsequent long-segment posterior fixation and fusion is still needed. Due to the biomechanical stresses of correcting ASD, multiple rods with multi-pelvic fixation have been used to load-share across these complex constructs [29,30]. Robotics systems allow for the preoperative planning of these complex designs, which further allow for their subsequent execution in the operating room [16]. Ten of twelve patients underwent multi-rod and multi-pelvic fixation via an MIS approach which was only feasible through the ability to preoperatively design and plan these constructs. Our workflow yielded a minimally invasive screw placement accuracy of 98.2% with three lateral breaches and one medial breach identified on intraoperative CT that were subsequently repositioned without neurological deficits or other complications.

A purported benefit of MIS approaches for deformity correction is the preservation of the entirety of the proximal soft tissue envelope during placement of all instrumentation for the prevention of PJK and PJF. Still, we observed two patients who experienced PJF requiring reoperation and proximal extension of their constructs and one patient with radiographic PJK after a fall, highlighting the complex etiologies of this postoperative complication. The two patients who experienced PJF (patients 2 and 3) were early in our series, both of whom underwent transdiscal multilevel stabilization screws (MLSS) [31,32]. With early PJK and subsequent PJF requiring reoperation, we have since abandoned this technique. Ishihara et al. have demonstrated more promising results in PJK prevention, focusing on the proximal screws at the UIV by increasing the pedicle screw angle such that there is a more anatomic approach trajectory toward the anterior inferior vertebral body rather than parallel to the endplate [33]. In addition to longer screw length, this allowed for increased pullout strength at the UIV to prevent the screw from backing out and affecting the proximal disc space that could continue to propagate PJK. They also noted that further kyphosis contouring of the proximal rod to match postoperative reciprocal change in the thoracic spine showed a reduction in PJK and mechanical complications as well. Longer follow-up with a larger cohort over time will be needed to show if there are other mechanisms at play for the observed mechanical complications in our experience.

Lastly, because minimally invasive deformity correction relies on imaging and visualization of the anatomy, the ionizing radiation exposure of both surgeon and patient remains a concern [34]. Patients in our series inevitably underwent a total of three CT scans of their thoracolumbar to lumbosacral spine: (1) prior to surgery for preoperative planning and full understanding of the deformity, (2) between Stage 1 and Stage 2 after interbodies were placed for minimally invasive pedicle screw placement with the robotic software platform, and (3) an intraoperative confirmation CT scan during Stage 2 to confirm that all screws are in appropriate position. While risks of radiation-induced cancers vary substantially by age and gender at the time of exposure, with the risks being lowest in older patients that are usually the population requiring adult spinal deformity correction [35], this patient radiation exposure provides a significant caveat for minimally invasive surgeries as well as an opportunity for further technological development through improved software registration to obviate the need for an interstage CT and lower dose imaging for intraoperative confirmation scans.

We present here the first case series to our knowledge describing in detail the application of robotics systems for minimally invasive adult spinal deformity surgery with posterior instrumentation and fusion to the lower thoracic spine.

### Limitations

Our study has several limitations known to retrospective case series, which include electronic charting errors, inaccuracies in radiographic measurements, and selection bias. This was also a single-center and single-surgeon study which precludes at this time a broader conclusion on its applicability across other clinical sites. Because many patients early in our series did not have preoperative patient-report outcome measures (PROMs), we were unable to discuss these results. As such, this paper relies on prior work showing that realignment and correction of radiographic targets correlates with improved PROMs and clinical outcomes measures [36]. Given the preliminary initial experience of this technique, the described study cohort is small and further research is still needed with larger patient populations across multiple centers to demonstrate its external validity.

## 5. Conclusions

We present here our series of patients with robot-assisted MIS deformity correction for ASD with proximal instrumentation in the lower thoracic spine, the majority of which required multi-rod and multi-pelvic fixation. We demonstrate in this report that the preoperative design and optimization of these large constructs provided a practical intraoperative surgical workflow which yielded favorable radiologic correction parameters and complication profiles when compared to robot-assisted open techniques. Mean operative time was 5 h 47 min for a mean fusion length of 9.9 levels, highlighting the proficiency of robotic assistance. All patients showed improvements in radiographic parameters and benefited from a low perioperative complication profile consistent with MIS approaches. The present study, to our knowledge, is the first series describing in detail the use of robotics systems for long-segment minimally invasive adult spinal deformity surgery. The increased applicability of these techniques and approaches will elucidate technique variability among surgical centers, in an effort to expand MIS approaches to more patients with ASD.

## Figures and Tables

**Table 1:** Summary of baseline characteristics of 12 patients included.

Sex	Number (%)
Male	2 (17)
Female	10 (83)
**Age**	68.6 (range 60–77)
**BMI**	28.1 (range 17.1–38.8)
**Diagnosis**	
Sagittal imbalance	4 (33)
Adult degenerative scoliosis	8 (67)
**Fusion Extent**	
Interbody cages	3.9 (range 2–6)
Levels instrumented	9.9 (range 8–11)
Pedicle screws * Rods	18.7 (range 13–24) 3.3 (range 2–4)

* includes S2-alar-iliac and iliac screws.

**Table 2:** Data regarding patient demographics and treatment.

Pt	Age/ Sex	BMI	Procedure	Levels Fused	Rods/Iliac Fixation	Operative Time * (hh:mm)	Discharge	LOS (d)	Follow-Up (m)
1	71F	38.5	L3-S1 OLIF, T11-ilium PSF	9	3/3	4:02	Home	5	38
2	64F	18.8	L4-S1 OLIF, T10-ilium PSF	10	4/4	4:46	ARU	4	42
3	72F	32.3	L2-S1 OLIF, T10-ilium PSF	10	4/4	5:24	ARU	5	30
4	76M	22.7	T12-L4 OLIF, T9-ilium PSF	11	4/4	5:48	Home	6	31
5	60F	38.8	T12-S1 OLIF, T9-ilium PSF	11	4/4	6:22	Home	10 **	20
6	77F	17.1	L1-S1 OLIF, T12-ilium PSF	8	2/2	4:21	ARU	8	19
7	71F	23.8	T12-L1, L4-S1 OLIF, T10-ilium PSF	10	2/2	6:45	Home	5	19
8	71F	26.5	L3-S1 OLIF, T10-ilium PSF	10	3/3	5:42	Home	4	17
9	69F	28.5	L1-S1 OLIF, T9-ilium PSF	11	3/3	6:42	Home	6	17
10	71M	29.7	L1-S1 OLIF, T11-ilium PSF	9	4/4	5:38	ARU	7	13
11	56F	22.6	L4-S1 OLIF, T10-ilium PSF	10	3/3	6:38	Home	5	8
12	65F	37.6	L2-4 ACR, L5-S1 OLIF, T10-ilium	10	3/3	7:22	ARU	5	6

* Posterior stage for screw fixation. ** Due to aspiration pneumonia treatment and persistent drug fevers. Pt = patient; M = male; F = female; ACR = anterior column realignment; OLIF = oblique lumbar interbody fusion; PSF = posterior spinal fixation; LOS = length of stay; d = days; m = months; mm = millimeter; ARU = acute rehabilitation unit.

**Table 3:** Data regarding patient preoperative and postoperative radiographic parameters.

Pt	SVA (cm)		PT (°)		PI-LL (°)		Coronal Cobb (°)	
1	3.8	2.1	34	22	20	7	54	32
2	6.8	0.8	24	15	16	8	-	-
3	9.6	5.1	35	22	29	10	-	-
4	10.4	3.4	38	21	45	7	-	-
5	5.6	4.8	44	21	48	8	47	4
6	21	0.7	30	18	43	−4	37	15
7	−0.9	1.1	26	19	−12	−7	29	3
8	7.6	5.7	29	22	26	6	38	12
9	7.6	−2.9	19	18	10	−3	41	15
10	3.2	2.5	18	12	1	−7	32	13
11	−1.9	2.6	26	16	9	1	50	16
12	20.4	5.5	28	15	55	1	-	-

## Data Availability

The raw data supporting the conclusions of this article will be made available by the authors on request.
